# Post-exposure administration of chimeric antibody protects mice against European, Siberian, and Far-Eastern subtypes of tick-borne encephalitis virus

**DOI:** 10.1371/journal.pone.0215075

**Published:** 2019-04-08

**Authors:** Andrey L. Matveev, Irina V. Kozlova, Oleg V. Stronin, Yana A. Khlusevich, Elena K. Doroshchenko, Ivan K. Baykov, Oksana V. Lisak, Ljudmila A. Emelyanova, Olga V. Suntsova, Vera A. Matveeva, Julia S. Savinova, Nina V. Tikunova

**Affiliations:** 1 Institute of Chemical Biology and Fundamental Medicine, Siberian Branch of Russian Academy of Sciences, Novosibirsk, Russia; 2 Federal State Public Scientific Institution «Scientific Centre for Family Health and Human Reproduction Problems», Siberian Branch of Russian Academy of Sciences, Irkutsk, Russia; 3 Novosibirsk State University, Novosibirsk, Russia; New York State Department of Health, UNITED STATES

## Abstract

Tick-borne encephalitis virus (TBEV) is the most important tick-transmitted pathogen. It belongs to the *Flaviviridae* family and causes severe human neuroinfections. In this study, protective efficacy of the chimeric antibody chFVN145 was examined in mice infected with strains belonging to the Far-Eastern, European, and Siberian subtypes of TBEV, and the antibody showed clear therapeutic efficacy when it was administered once one, two, or three days after infection. The efficacy was independent of the TBEV strain used to infect the mice; however, the survival rate of the mice was dependent on the dose of TBEV and of the antibody. No enhancement of TBEV infection was observed when the mice were treated with non-protective doses of chFVN145. Using a panel of recombinant fragments of the TBEV glycoprotein E, the neutralizing epitope for chFVN145 was localized in domain III of the TBEV glycoprotein E, in a region between amino acid residues 301 and 359. In addition, three potential sites responsible for binding with chFVN145 were determined using peptide phage display libraries, and 3D modeling demonstrated that the sites do not contact the fusion loop and, hence, their binding with chFVN145 does not result in increased attachment of TBEV to target cells.

## Introduction

Tick-borne encephalitis virus (TBEV), a positive-sense single-stranded RNA virus from the *Flaviviridae* family, is a causative agent of one of the most severe human neuroinfections [1–3]. TBEV is mostly transmitted via bites by *Ixodes* ticks that inhabit the forested areas of Eurasia from Western Europe to the Far East and from the Scandinavian peninsula to the Mediterranean [4]. Three main subtypes of TBEV are currently recognized: the Far-Eastern (TBEV-FE), European (TBEV-Eu), and Siberian (TBEV-Sib) subtypes [5–7]. TBEV-FE is considered to cause the most severe disease, while TBEV-Eu causes mostly mild infections [6,8–10]. Tick-borne encephalitis (TBE) is documented in many European countries, Russia, China, Mongolia, and Kazakhstan, and, in the past several years, the highest incidence of the disease was recorded in Russia, Slovenia, and the Baltic states [11]. Although most human cases of TBE are asymptomatic, TBEV can cause severe TBE, which is often lethal [3,6,12]. Several vaccines for the prevention of TBE in adults and children are currently available in Europe, Russia, and China. These vaccines are safe, highly immunogenic, and efficient; however, vaccination coverage is low in many regions, which leads to a substantially higher frequency of TBE cases in these regions. Moreover, TBEV vaccine breakthrough cases have been recorded in several countries [13–15].

In most endemic countries, there are no specific preparations for the treatment of TBE. Previously, specific anti-TBE serum immune globulin Encegam (FSME-Bulin) was used in European countries; however, the use of Encegam was suspended due to concerns regarding possible enhancement of TBE after its administration. In Russia, where the most severe and lethal cases of TBE have been recorded, specific anti-TBE serum immunoglobulin (anti-TBE-Ig) is applied for post-exposure prophylaxis and treatment of TBE [3,16]. Anti-TBE-Ig can decrease the severity of the disease [17]; however, like other preparations derived from the donor’s blood, it has disadvantages including a limited number of plasma from donors vaccinated against TBE; insufficient standardization of original plasma and, as a consequence, insufficient standardization of the preparation; increased risk of contamination with non-detectable pathogens. In this regard, the development of alternative anti-TBEV immunological preparations is required.

Previously, several anti-TBEV chimeric antibodies were constructed [18,19] based on variable domains of mouse monoclonal antibodies against glycoprotein E of TBEV-FE. Two chimeric antibodies were able to neutralize TBEV infectivity *in vitro* and one of them protected mice that were infected with TBEV-Eu [18]. In this study, the therapeutic potency of the chimeric antibody chFVN145 was examined in mice infected with strains belonging to the Far-Eastern, Siberian, and European subtypes of TBEV and treated once with chFVN145 one, two, and three days after infection. In addition, the neutralizing epitope of chFVN145 was determined.

## Materials and methods

### Viruses

TBEV strains Sofjin and Vasilchenko, prototype Russian strains of TBEV-FE and TBEV-Sib, respectively, were obtained from the repository at the Federal State Public Scientific Institution «Scientific Centre for Family Health and Human Reproduction Problems» (Collection # 478258, http://www.ckp-rf.ru, Irkutsk, Russia); the TBEV strain Absettarov (TBEV-Eu) was obtained from the repository at the Tomsk branch of the Federal State Unitary Company “Microgen” (Tomsk, Russia). All experiments with live TBEV were conducted under BSL-3 conditions.

### Development of chFVN145

To develop chFVN145, DNA fragments encoding variable domains of heavy and light chains (VH and VL) of a mouse monoclonal antibody against glycoprotein E of TBEV were amplified by RT-PCR using previously designed primers [18] and total RNA from hybridoma cell line 14D5 [20]. A stable clone producing chFVN145 was obtained as described previously [21]. Briefly, the DNA fragment encoding VH was restricted by *Not*I and *Pme*I endonucleases and inserted into the *Not*I/*Pme*I-digested plasmid pBiFRT designed previously [21] to generate the pBiFRT-VH145 plasmid. Then, the VL-encoding DNA fragment cleaved by *EcoR*V and *Hind*III was cloned into the *EcoR*V/*Hind*III-digested plasmid pBiFRT-VH145. The resulting plasmid, pBiFRT-145, and the plasmid pOG44 (Life Technologies, Carlsbad, CA) were co-transfected into suspension CHO-S/FRT cells [22] using PEIPro (Polyplus-transfection SA, Illkirch, France) for site-directed genomic integration. Two days after transfection, the culture medium was replaced with the selective medium CD OptiCHO (Life Technologies) containing 50 μg/mL hygromycin B, and the stable clone CHO-S/FRT/FVN145 was selected according to the manufacturer's instructions. CHO-S/FRT/FVN145 cells were cultivated in CD FortiCHO medium (Life Technologies) with addition of 4 mM glutamine (Biolot, St. Petersburg, Russia) and 3 mM glucose (Biosyntez, Penza, Russia) every three days. To purify chFVN145, cells and debris were harvested by centrifugation; the supernatant was filtered through a 0.22-μm PES capsule filter (Millipore, Burlington, MA) and loaded onto a 4-mL Protein A-sepharose column. ChFVN145 was eluted in 0.1 M citric buffer (pH 3.0). The antibody was concentrated and buffer-exchanged against phosphate buffer saline (PBS, pH 7.4) using an Amicon Ultra-4 50 kDa filter (Millipore, USA), sterilized by filtration through a 0.22 μm filter (Millipore, USA), and stored at a concentration of 2 mg/ml at 4°C.

### Affinity constant measurement

The kinetics for chFVN145 binding with TBEV glycoprotein E was determined by a surface plasmon resonance (SPR) method using a ProteOn XPR36 System (Bio-Rad, USA). Recombinant protein rE [23] was immobilized onto vertical channel L1 of GLC sensor chip at a 70 response units (RU) level. Serial dilutions of chFVN145 were analyzed starting from the lowest concentration (1 nM, 3 nM, 9 nM, 27 nM, and 81 nM) at a flow rate of 25 μl/min. Vertical channel L2 was used as a reference channel. Binding experiments were performed in triplicate; chip surface was regenerated with 100 mM citric acid. Global analysis of experimental data based on a single-site or a heterogeneous analyte models was performed using the ProteOn Manager v. 3.1.0 software. The affinity constant was calculated as K_D_ = k_d_/k_a_.

### Animal studies

BALB/c mice were purchased from the animal care facility in the Federal State Research Center of Virology and Biotechnology “Vector” (Koltsovo, Russia). Animals were housed under normal light-dark cycle; water and food were provided *ad libitum*. Before the experiments, LD_50_ was determined for each viral stock. Groups of three-week old mice (n = 10 in each group) were infected intraperitoneally with serial ten-fold dilutions of a viral stock in 0.9% NaCl and LD_50_ for each stock was calculated by the Reed–Muench method [24]. Then, viral stocks were aliquoted and stored at -80°C.

To examine the protective efficacy of antibody preparations, three-week old BALB/c mice (9–10 g) were infected intraperitoneally with TBEV at the appropriate dose in 0.2 mL. One, two, or three days after infection, the mice were treated once intramuscularly with chFVN145, a commercial preparation of human anti-TBE immunoglobulin (anti-TBE-Ig) produced from donor blood (lot #608, 10%, hemagglutination titer 1:160, Virion, Russia), or 0.9% NaCl in a volume of 100 μL. Preparations of chFVN145 and anti-TBE-Ig were diluted in 0.9% NaCl. Each experimental group of mice included 8–10 animals. Mice were monitored at least twice a day for clinical signs of the disease (paresis and/or paralysis), which resulted in euthanasia using an overdose of isoflurane. The mice were observed for 21 days after infection, and the survival rate was recorded. In addition, the mean survival time (MST) was determined as the period between the infection and animal death; the MST of mice that survived until the end of the experiment was 21 days. To consider the effect of storage and freezing-thawing on the viral stock, actual infectious dose (in LD_50_) was additionally determined in each experiment based on the method described previously [24] using four groups of mice, six animals in each group.

All animal procedures were carried out in accordance with the recommendations for the protection of animals used for scientific purposes (EU Directive 2010/63/EU). All experiments with animals were approved by the local bioethics committee of the Federal State Public Scientific Institution «Scientific Centre for Family Health and Human Reproduction Problems».

### Expression of recombinant E proteins

Recombinant variants of TBEV glycoprotein E, rED1+2 (domains I + II, amino acid residues, aa 1–302) and rED3_301 (domain III, aa 301–397) were designed previously [23]. Breifly, gene fragments encoding these recombinant proteins were derived from the Sofjin-Ru strain (GenBank accession number AEP20480), inserted into the pHEN2 plasmid, and produced in *Escherichia coli* HB2151 cells.

The recombinant proteins rED3delA+ and rED3delD+, which included fragments of domain III of glycoprotein E (aa 301–359 and aa 356–397, respectively), were obtained in this study using the expression vector pHEN2. DNA fragments encoding rED3delA+ and rED3delD+ were amplified by PCR using cDNA of the same strain (Sofjin-Ru) as a template and two pairs of the primers: 5′-GCGCCATGGCCGGCGGTGGCTCGGGTCTTACATACACAATGTGCG-3′ and 5′-TTAGCGGCCGCTGTTATCAACATGGCCACGTTCACATCCG-3′ for rED3delA+ and 5′-GCGCCATGGCCGGCGGTGGCTCGATGTTGATAACACCCAACCCC-3′ and 5′-TTAGCGGCCGCTTAGTGATGGTGATGATGATGACTCCCTTTTTGGAACCATTG-3′ for rED3delD+, respectively. The resulting PCR fragments were cleaved with *Nco*I and *Not*I and ligated independently into *Nco*I/*Not*I-digested pHEN2. *E*. *coli* HB2151 cells were transformed with the resulting plasmids, plated onto agar with 50 μg/mL ampicillin and 20 μg/mL isopropyl-β-D-1-thiogalactopyranoside and cultivated. Individual colonies of *E*. *coli* producing rED3delA+ and rED3delD+ were screened by PCR using the same primers, and their ability to produce the recombinant proteins was confirmed using 12.5% SDS-PAGE.

### Western blot analysis

*E*. *coli* cells producing the recombinant proteins rED1+2, rED3_301, rED3delA+, and rED3delD+ were harvested by centrifugation and lysed with a buffer (50 mM Tris-HCl, pH 6.8, 200 mM dithiothreitol, and 4% sodium dodecyl sulfate). The lysates were fractioned using 12.5% SDS-PAGE and transferred onto a nitrocellulose membrane (Bio-Rad, Hercules, CA). After blocking with 5% skimmed milk in phosphate-buffered saline (PBS) with 0.05% Tween 20 (PBST), the membrane was incubated with 5 μg/mL chFVN145 at 37°C for 1 h. Immune complexes were detected using alkaline phosphatase-conjugated goat anti-human IgG (Sigma-Aldrich, St. Louis, MO) and visualized by a mixture of nitro blue tetrazolium (Amresco, Radnor, PA) and 5-bromo-4-chloro-3-indolylphosphate (Roche, Basel, Switzerland) for 20 min.

### Epitope mapping

Selection of specific peptides exposed on the surface of bacteriophages from the phage display libraries PhD-12 and PhD-C7C (New England Biolabs, Ipswich, MA) was carried out as described previously [21]. Briefly, approximately 10^11^ phage particles from each library were pre-incubated with a non-specific mock mouse antibody and added to the wells of 96-well microtiter plates coated with 200 ng chFVN145 in PBS, pH 7.4, and incubated for one hour at 37°C. Then, unbound phage particles were washed away with PBST, and bound phages were eluted with 100 μg/mL chFVN145. The eluted phages were used for the second round of biopanning, which was carried out using plates coated with 20 ng chFVN145. Phage particles that were eluted after the second round were used to transfect *E*. *coli* ER2738 cells. Phages with the selected exposed peptides were isolated from individual plaques and assayed for their binding to chFVN145 by ELISA. For indirect ELISA, the wells of 96-well microtiter plates were coated with 20 ng of chFVN145 or 3% bovine serum albumin in PBS, pH 7.4. After blocking, individual selected phages were diluted in PBST to yield ~10^10^ colony forming units (CFU) and added to the wells. Indirect ELISA was performed with anti-M13 polyclonal rabbit antibodies followed by alkaline phosphatase-conjugated anti-rabbit IgG (ICN) and stained with p-nitrophenyl phosphate.

### Statistics

The data were analyzed using Microsoft Excel software and are expressed as mean values ± standard error of the mean (SEM). Comparisons were performed using the log-rank test. Data were analyzed using the on-line service available at https://www.evanmiller.org/ab-testing/survival-curves.html. P < 0.05 was considered to indicate statistical significance.

## Results

### Production and characterization of chFVN145

**Fig 1:**
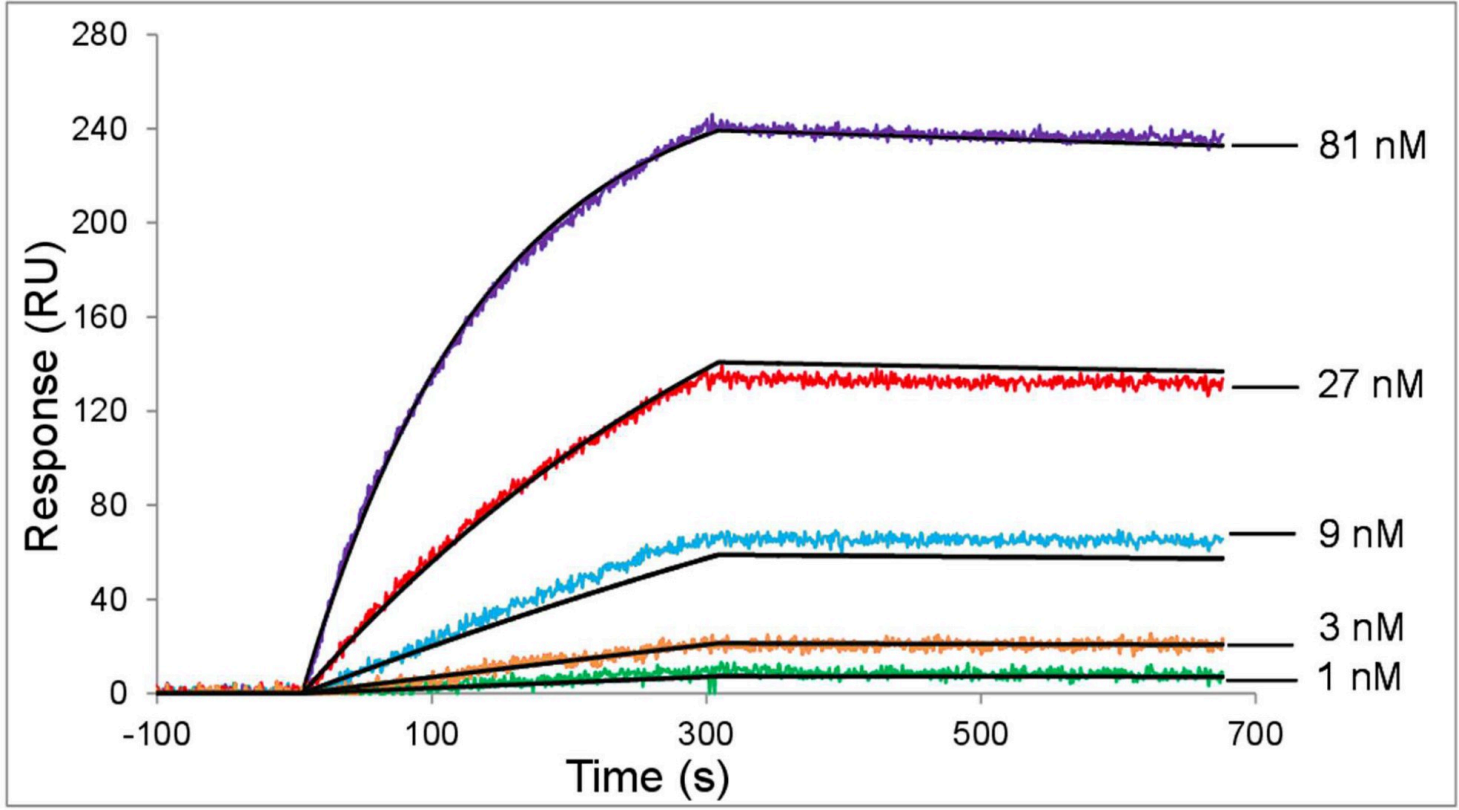
The affinity of chFVN145 for recombinant protein E of TBEV. Serial three-fold dilutions of chFVN145 starting from 81 nM were used as the analyte. Fitted traces are depicted as black lines. A global analysis of the interaction demonstrated a good quality fit and the affinity constant, calculated as K_D_ = k_d_/k_a_, was 1.5 ± 0.2 nM.

To produce chFVN145, the stable clone CHO-S/FRT/FVN145 was developed based on previously obtained cell line CHO-S/FRT [21] and the plasmid, pBiFRT-145, in which variable regions were connected to the constant regions of human IgG kappa. CHO-S/FRT/FVN145 cells were cultivated and chFVN145 was purified following procedures described in the Material and Methods section. The purified chFVN145 was examined by SDS-PAGE and western blotting. To analyze the binding affinity of chFVN145, a label-free biosensor assay was used. A global analysis of interaction between chFVN145 and recombinant protein rE demonstrated a good quality fit and the affinity constant was calculated as K_D_ = (1.5 ± 0.2) × 10^−9^ M (Fig 1).

### chFVN145 post-exposure prophylaxis of mice infected with TBEV-Eu, TBEV-FE, or TBEV-Sib strains

**Fig 2:**
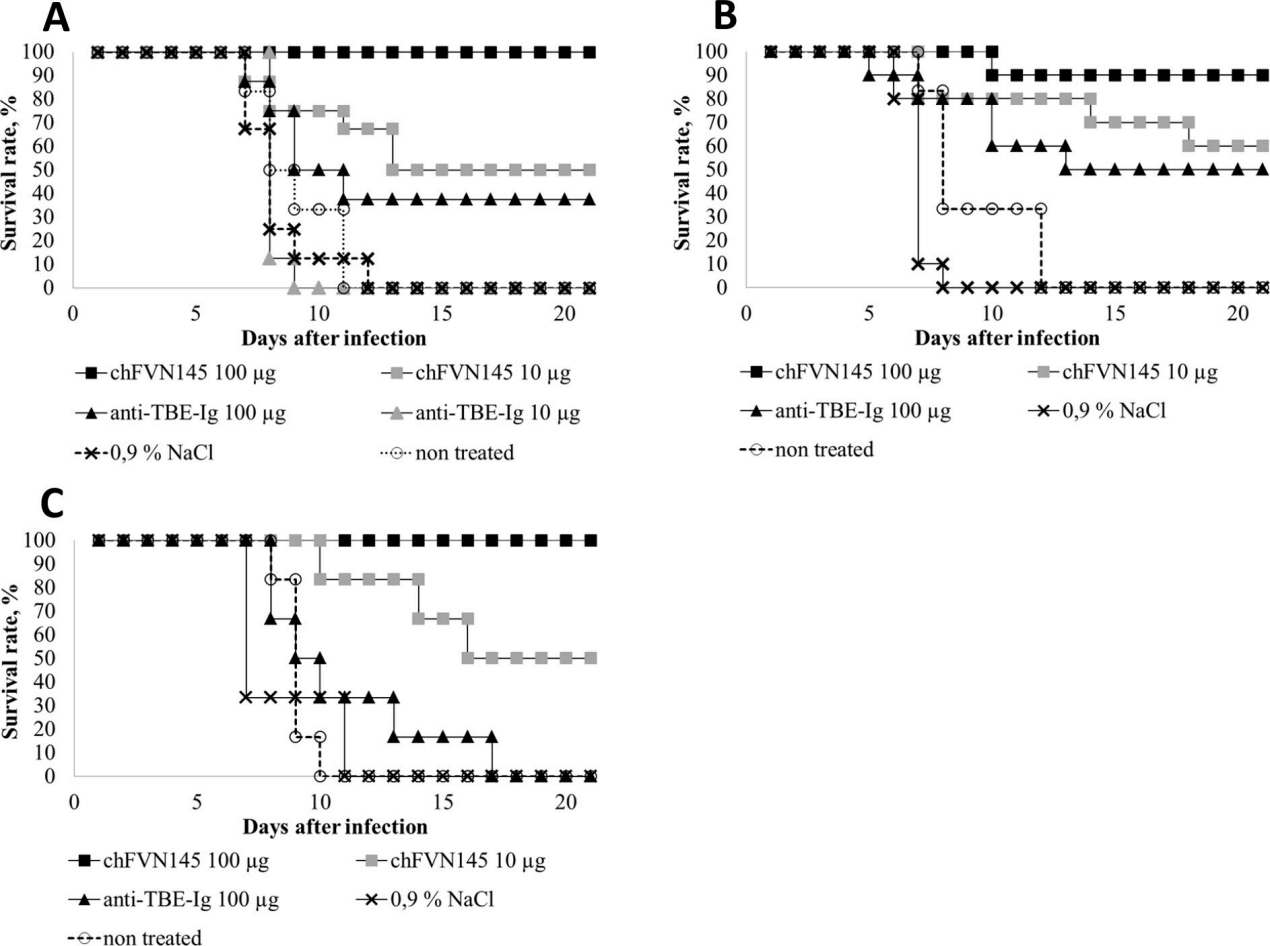
Post-exposure protection of mice infected with various subtypes of TBEV. BALB/c mice (10–11 g) were treated (i.v.) with chFVN145 or anti-TBE-Ig at the indicated doses one day after infection with (A) 159 LD50 TBEV-Eu, (B) 231 LD50 TBEV-FE, and (C) 251 LD50 TBEV-Sib.

Six groups of BALB/c mice were challenged by intraperitoneal injection of a target dose of TBEV-Eu 159 LD_50_. One day after infection, the first group of mice received chFVN145 at a dose of 100 μg/mouse (high dose) and the second group at a dose of 10 μg/mouse (low dose), whereas two other groups of animals were treated with anti-TBE-Ig at doses of 100 μg and 10 μg per mouse, respectively. The fifth group of mice was administered 0.9% NaCl, while a group of non-treated mice was used as the control group. Post-exposure administration of chFVN145 at target doses of 100 μg and 10 μg per mouse resulted in 100% (8/8) and 50% (4/8) survival rates, respectively (Fig 2A). Protective efficacy of anti-TBE-Ig at the high dose was lower than that of chFVN145 (37.5%, 3/8); no protection was observed when anti-TBE-Ig was used at the low dose (Fig 2A).

To test the protective efficacy of chFVN145 against TBEV-FE and TBEV-Sib, similar experiments were carried out when mice were challenged with the TBEV strains Sofjin and Vasilchenko at doses of 231 LD_50_ and 251 LD_50_, respectively. One day after infection, mice in each group were treated once with chFVN145 (100 μg and 10 μg per mouse), anti-TBE-Ig at a dose of 100 μg/mouse, or 0.9% NaCl. Clear protection against TBEV infection in the treated mice was observed (Fig 2B and 2C). One injection of 100 μg/mouse chFVN145 resulted in 90% (9/10) survival rate in mice infected with TBEV-FE and 100% (8/8) in mice challenged with TBEV-Sib. When mice received 10 μg chFVN145, the survival rates were 60% (6/10) and 50% (4/8) for TBEV-FE and TBEV-Sib, respectively (Fig 2B and 2C). Administration of anti-TBE-Ig provided protection in 50% (5/10) mice infected with TBEV-FE and was not effective in mice infected with TBEV-Sib (Fig 2C). However, the MST of animals from this group was not decreased compared to that of the controls, namely 9.8 ± 3.5 vs. 7.3 ± 2.1 in the non-treated mice.

### Therapeutic efficacy of chFVN145

**Fig 3:**
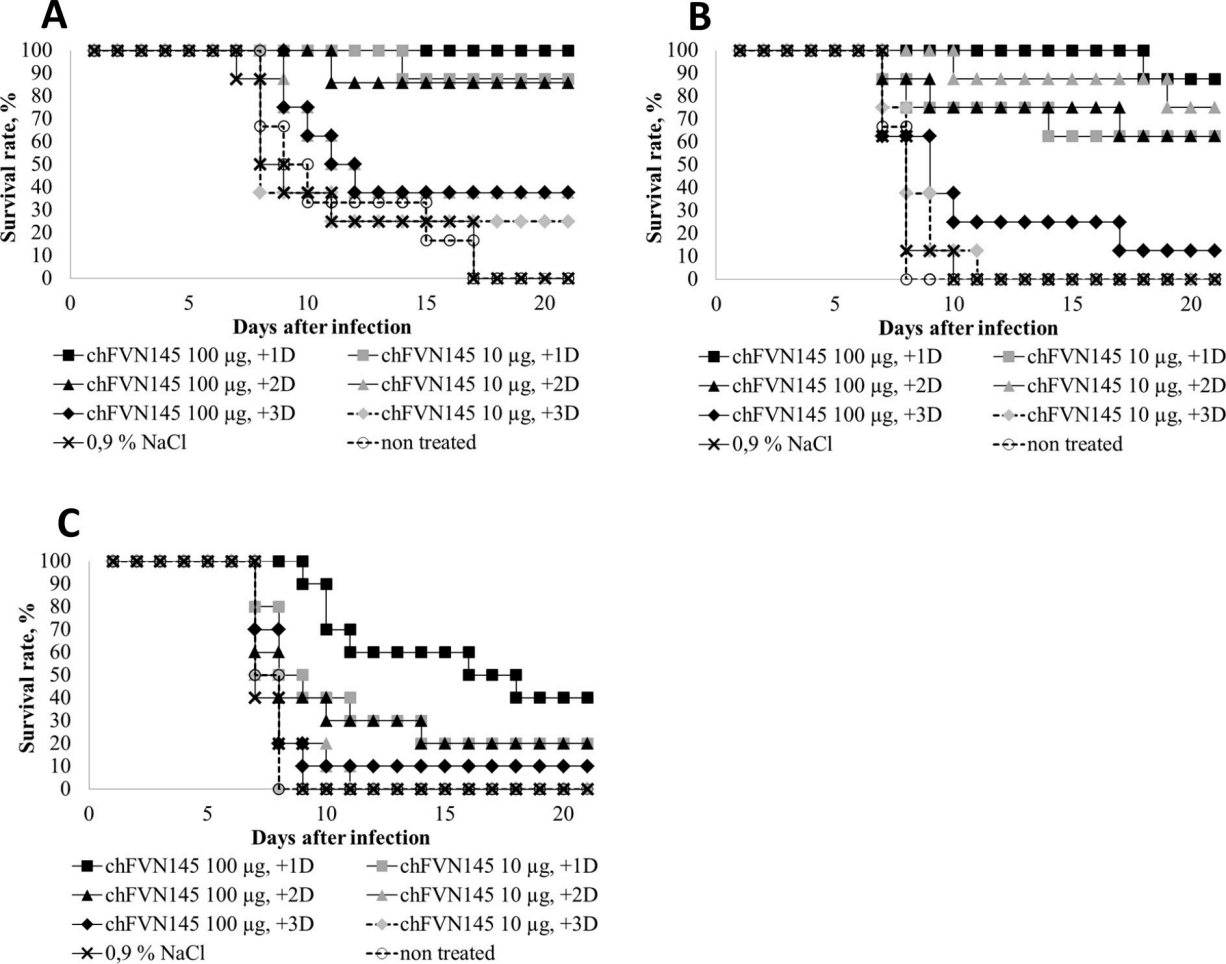
Therapeutic efficacy of chFVN145 in mice infected with different subtypes of TBEV. BALB/c mice (10–11 g) were treated (i.v.) once with chFVN145 or anti-TBE-Ig one, two, and three days after infection with (A) 20 LD50 TBEV-Eu, (B) 501 LD50 TBEV-FE, and (C) 3981 LD50 TBEV-Sib.

To assess the therapeutic efficacy of chFVN145, mice infected with TBEV-Eu were treated once with the antibody (at 100 μg and 10 μg per mouse) one, two, or three days after infection (designated +1, +2, and +3). Administration of 100 μg of chFVN145 showed good therapeutic effect and resulted in 100% (8/8), 85.7% (7/8), and 62.5% (5/8) survival rates in infected mice that received the antibody at days +1, +2, and +3, respectively (Fig 3A). Even treatment with the low dose of chFVN145 improved the survival rate of mice and resulted in a substantial increase of the MTS (20 ± 2.8, 13.5 ± 6.3, and 10.9 ± 6.3 at days +1, +2, and +3, respectively) when compared to the non-treated controls and mice injected with 0.9% NaCl, (10.2 ± 3.9 and 9.8 ± 4.5, respectively; Fig 3A).

Next, mice challenged with 501 LD_50_ TBEV-FE were administered chFVN145 at high and low doses at days +1, +2, and +3. Good therapeutic effect was observed at days +1 and +2 for both high and low doses, and only weak improvement in the survival rate was recorded on day +3 for the high dose (Fig 3B). However, even when mice were treated with chFVN145 at a dose of 10 μg/mouse on day +3 (100% mortality rate), the MST was not lower than that of mice received 0.9% NaCl and non-treated controls (7.4 ± 1.3 vs. 6.9 ± 1.0 and 6.6 ± 0.5, respectively), indicating that the course of the disease in this group of mice was not more severe compared to controls.

Finally, the therapeutic potency of chFVN145 was examined when mice were infected with TBEV-Sib. In this experiment, mice were challenged with a high lethal dose of TBEV strain Vasilchenko, 3981 LD_50_. Even in this case, administration of chFVN145 increased the MST of mice treated at days +1, +2, or +3 compared to that of the controls (Fig 3C). Expectedly, the survival rate of mice that received 100 μg of chFVN145 was higher than that of mice treated with the antibody at a low dose (Fig 3C).

### Determination of non-protective doses of chFVN145

**Fig 4:**
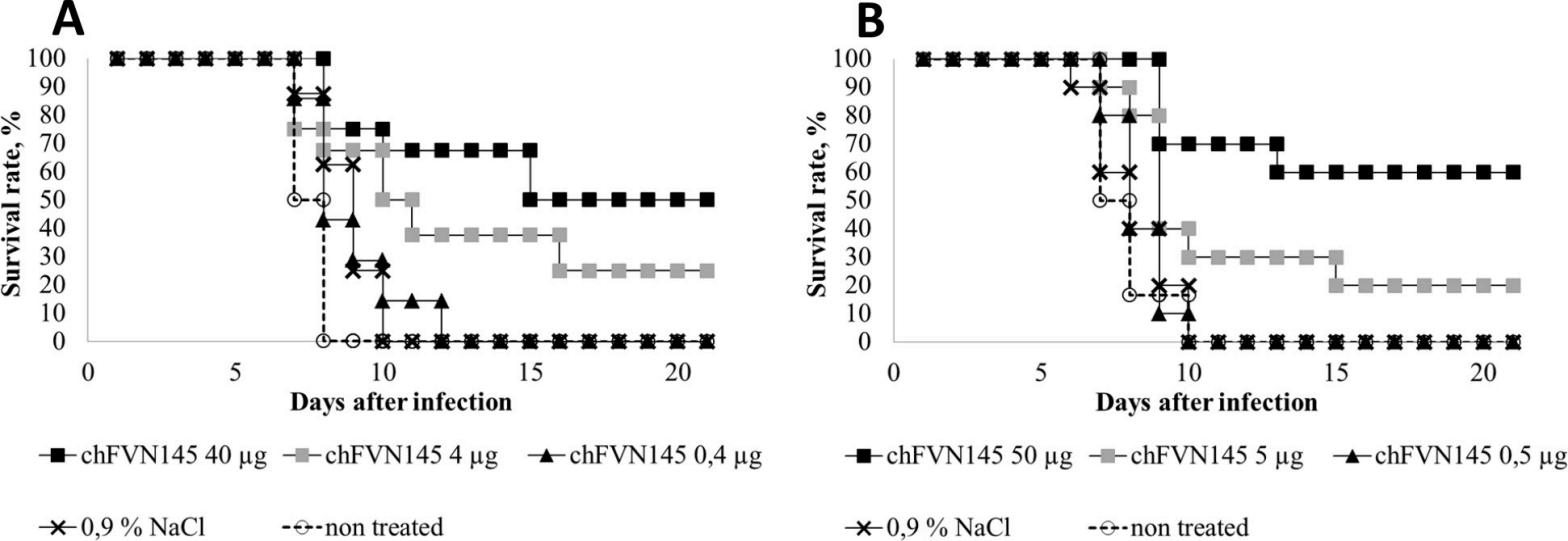
Determination of non-protective doses of chFVN145 doses. BALB/c mice (10–11 g) were treated (i.v.) with chFVN145 at the indicated doses one day after infection with (A) 199.5 LD50 TBEV and (B) 316 LD50 TBEV.

To examine whether administration of chFVN145 can enhance TBEV infection in the mouse model, a non-protective dose of the antibody was determined when the mice were treated one day after infection. For this, mice infected with 199.5 LD_50_ TBEV-Eu were administered chFVN145 at doses of 40 μg, 4 μg, or 0.4 μg per mouse. The survival rate was improved when the mice were treated with 40 μg/mouse and 4 μg/mouse, but not when mice received 0.4 μg of chFVN145: in that case, a mortality rate of 100% was observed (Fig 4A). The experiment was repeated with mice challenged with 316 LD_50_TBEV-FE (Fig 4B). Importantly, no significant difference was observed in the MST of mice that received non-protective doses of chFVN145 and the non-treated controls and mice administered 0.9% NaCl. Non-protective doses of chFVN145 were determined: approximately 0.5 μg/mouse for treatment at day +1, 10 μg/mouse at day +2, and 10–100 μg/mouse at day +3.

**Fig 5:**
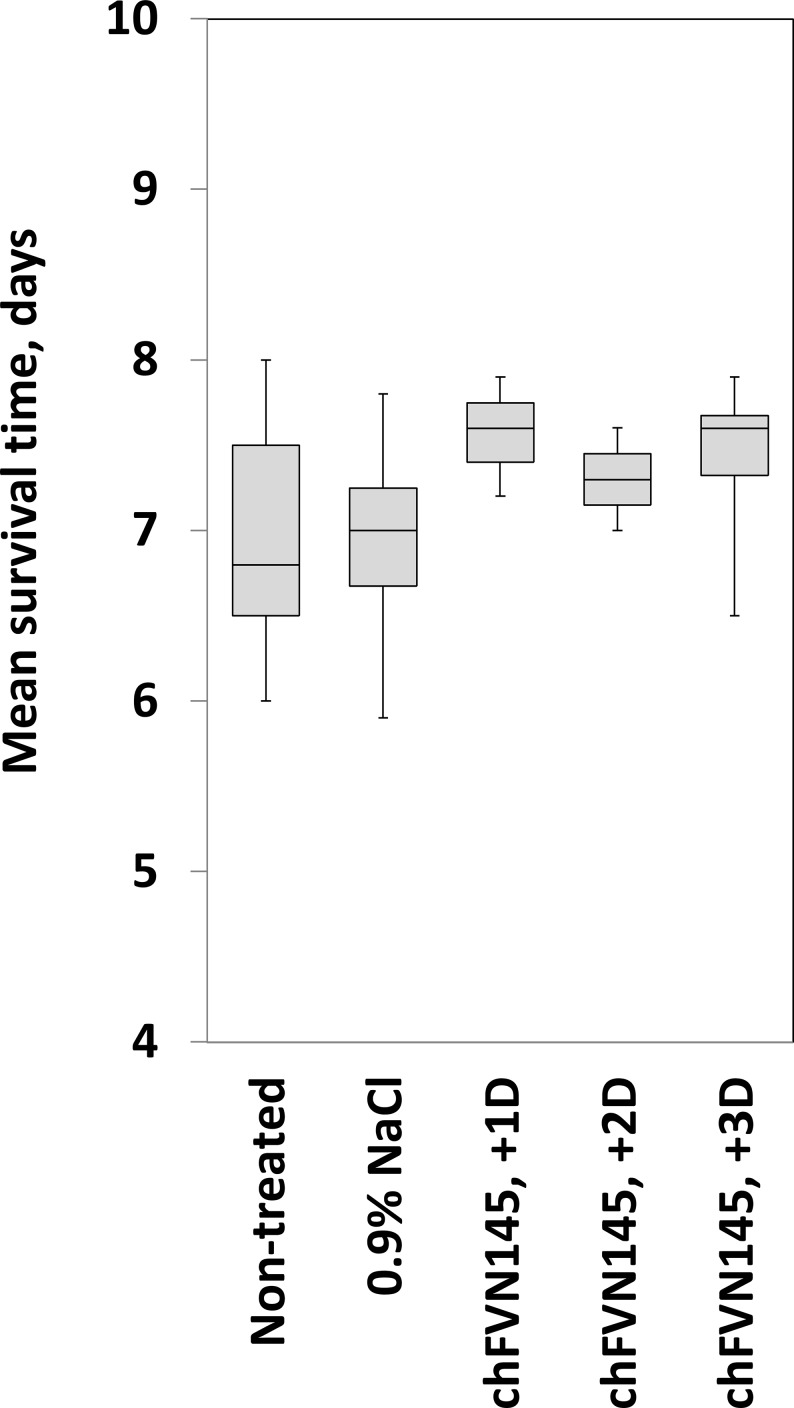
The mean survival time of mice treated with non-protective doses of chFVN145. The box-and-whisker plots represent the mean survival time of different groups of mice infected with TBEV with 100% mortality rate. Administration of non-protective doses did not decrease the survival rate and MST of treated mice compared to those of mice received 0.9% NaCl and non-treated controls independent of the timing of chFVN145 injection and TBEV strain used for infection.

Summarized data on the MST of mice from the groups with a mortality rate of 100%, including non-treated mice and mice that received chFVN145 or 0.9% NaCl showed that the MST of mice treated with chFVN145 at non-protective doses at days +1, +2, or +3 was comparable to or better than the MST of non-treated mice and mice received 0.9% NaCl, indicating that chFVN145 did not enhance TBEV infection (Fig 5). Importantly, the results were independent on the TBEV strain used for infection.

### Neutralizing epitope mapping

**Fig 6:**
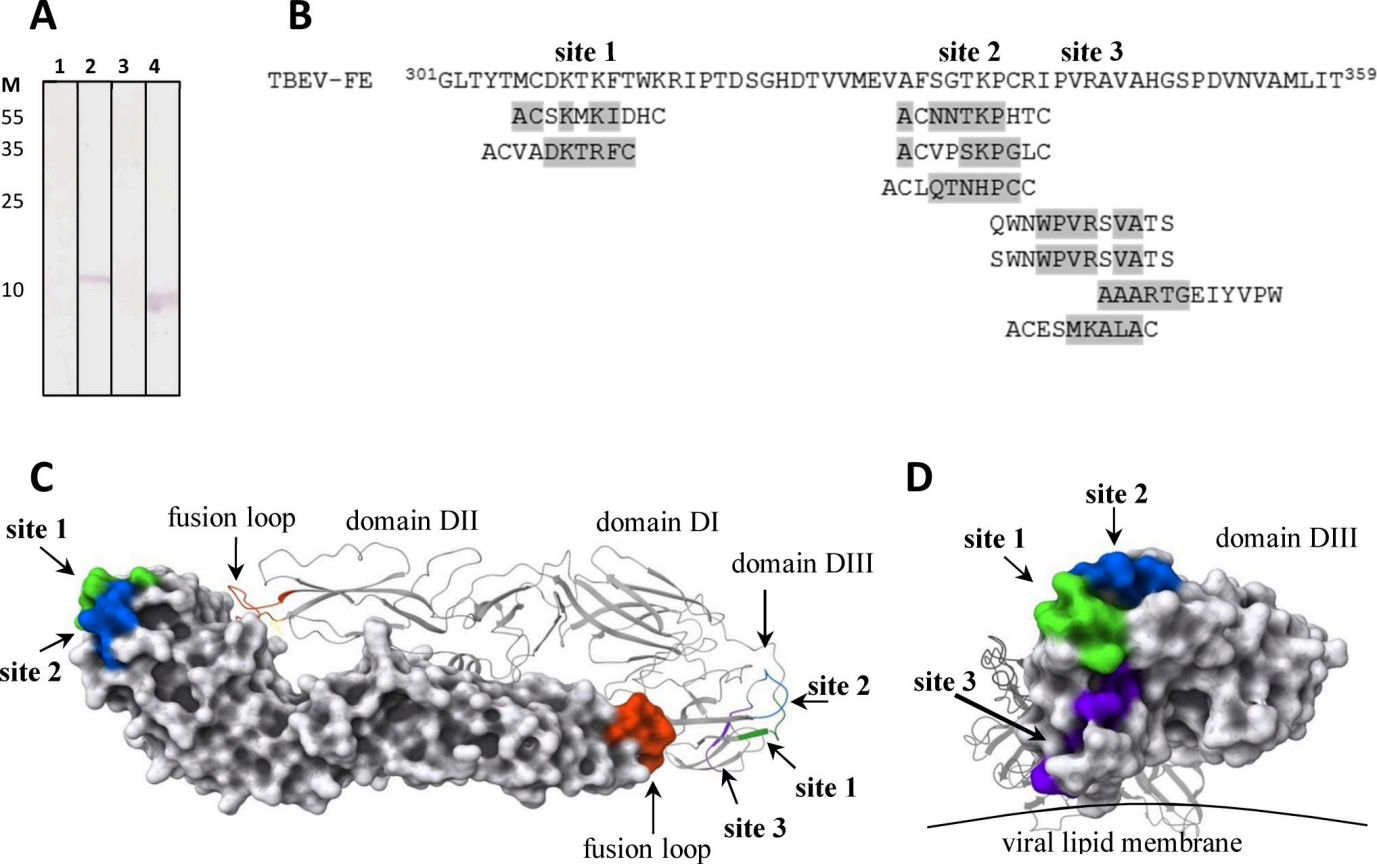
Epitope mapping of chFVN145. (A) Western blot analysis of lysates of E. coli cells producing rED1+2 (1), rED3_301 (2), rED3delD+ (3) and rED3delA+ (4), which were fractionated by SDS-PAGE (12.5%) and developed with chFVN145. Protein marker masses, in kilodaltons, are shown at the left side of the gel. (B) Results of phage display screening for peptides bound by chFVN145. Alignment of TBEV glycoprotein E homolog fragments and peptides, selected from PhD-12 and PhDC7C library TBEV protein E-like aa was marked in grey. (C) Ribbon and surface representations of TBEV glycoprotein E dimer structure (PDB 1svb) with indicated putative epitope sites. View from the outer surface of virion. (D) Ribbon and surface representations of proposed epitope sites, view from the DIII side of E glycoprotein. Molecular coordinates for the TBEV glycoprotein E structure (PDB 1svb) used in structural analysis were obtained from the Protein Data Bank then visualized using PyMol software (version 1.7.6.0).

For characterization of the neutralizing epitope of chFVN145, recombinant variants of TBEV glycoprotein E, including rED1+2 (aa 1–302), rED3_301 (aa 301–397), rED3delA+ (aa 301–359), and rED3delD+ (aa 356–397), were developed and produced in *E*. *coli* cells. Western blotting of lysates from *E*. *coli* cells producing the recombinant proteins showed that chFVN145 was specific to rED3_301, i.e., domain III, and bound to rED3delA+, but did not recognize rED3delD+ (Fig 6A).

To localize the neutralizing epitope, two random peptide libraries were screened using chFVN145. The PhD-12 library contains phages each with a random 12-mer peptide inserted into the N-terminus of the phage minor protein p3 and exposed on the phage surface. In the PhD-C7C library, an exposed 7-mer random peptide is flanked by cysteine residues, which form a peptide loop. Three different peptides with DIII-like sequences were selected from the PhD-12 library along with six peptides from the PhD-C7C library (Fig 6B). All identified sequences were localized within the rED3delA+ sequence, between aa 307 and 348. Three possible sites responsible for binding with chFVN145 were predicted: aa 307–313, aa 333–338, and aa 341–348, from which site 1 was located on the strand A, site 2 on the loop BC, and site 3 on the strand C and loop CD according to [25] (Fig 6C). Notably, sites 1 and 2 are located on the surface of domain III and available for binding, while site 3 is buried in the protein layer close to viral lipid membrane.

## Discussion

In this study, the therapeutic efficacy of the chimeric antibody chFVN145 was investigated in mice infected with various subtypes of TBEV. In previous experiments, ch14D5a purified from culture medium after transient expression in CHO-K1 cells showed protective efficacy against lethal infection of BALB/c mice with the TBEV strain Absettarov (TBEV-Eu) when ch14D5a was administered one day after infection [18]. A similar experiment was designed to test the ability of chFVN145 produced by the stable clone CHO-S/FRT/chFVN145 to protect mice challenged with the same TBEV strain. Commercially available anti-TBE-Ig, which is routinely applied for post-exposure prophylaxis and treatment of TBE in Russia [3,16], was used as a positive control. Since antibody preparations used in this study were diluted in 0.9% NaCl, mice treated with 0.9% NaCl were used as one of the controls. Clear dose-dependent efficacy of chFVN145 was observed, and the dose of 100 μg/mouse provided substantially better protection than the dose of 10 μg/mouse. The protective efficacy of chFVN145 was significantly higher than that of the anti-TBE-Ig that is probably due to the substantially lower amount of specific anti-TBEV immunoglobulins in the total protein in this preparation. The anti-TBE-Ig was not effective at a dose of 10 μg/mouse, resulting in the exclusion of this dose from further experiments.

As different TBEV subtypes occur in Eurasia, the protective efficacy of chFVN145 against virus strains belonging to TBEV-FE and TBEV-Sib was examined. The results of these experiments were similar to those obtained in mice infected with TBEV-Eu: chFVN145 demonstrated dose-dependent efficacy against both TBEV-FE and TBEV-Sib and was significantly more effective than anti-TBE-Ig. Importantly, despite the different pathogenicity of TBEV-Eu, TBEV-Sib, and TBEV-FE strains for humans, no substantial difference in the protective efficacy of chFVN145 against all three subtypes was observed in the mouse model.

Given this, the therapeutic efficacy of chFVN145 was assessed when mice were challenged with variable doses of target TBEV strains. The post-exposure window was expanded and mice were treated one, two, or three days after infection. The results indicated that survival rates were TBEV dose-dependent. When mice were infected at a dose of 20 LD50 (TBEV-Eu strain), the survival rate of mice administered chFVN145 at +1, +2, or +3 days substantially increased compared to that of non-treated mice and mice received 0.9% NaCl. When mice were infected at a dose of approximately 500 LD_50_ (TBEV-FE strain), good results were observed in mice that received chFVN145 on days +1 or +2. The survival rate of mice challenged with TBEV-Sib strain at a dose of approximately 4000 LD_50_ was substantially improved only when mice were treated one day after infection. However, even in the case of such a high infection dose, an improvement in the survival rate or the MST was observed when mice were given chFVN145 at days +2 or +3.

The ability of chFVN145 to augment TBEV infection in mice was specifically investigated due to concerns that some flavivirus-specific antibodies can induce antibody-dependent enhancement (ADE) of infection. This phenomenon was repeatedly observed for flaviviruses in *in vitro* and *in vivo* experiments [26–30]. Moreover, antibody-associated augmentation of disease has been recorded for dengue virus infection in humans [31–33]. ADE is mainly associated with the use of a sub-neutralizing concentration of antibodies [34,35]. In this study, non-protective doses of chFVN145 were determined: approximately 0.5 μg/mouse for treatment at day +1, 10 μg/mouse at day +2, and 10–100 μg/mouse at day +3. Importantly, administration of non-protective doses did not decrease the survival rate and MST of treated mice compared to those of mice received 0.9% NaCl and non-treated controls regardless of the timing of chFVN145 injection and the TBEV strain used for infection. Only one administration of chFVN145 was examined in this study, and further investigation is required for the assessment of the effects of multiple administrations of the antibody.

Recently, a novel mechanism of antibody-induced enhancement of flavivirus infection, which is based on the antibody-promoted conformational changes in glycoprotein E that result in an increased availability of the usually buried fusion loop (FL), has been described [36]. In this regard, the neutralizing epitope for chFVN145 was examined and potential sites on the surface of glycoprotein E responsible for binding with chFVN145 were determined using phage display. According to 3D modeling, these potential sites do not contact the FL, and, hence, chFVN145 binding cannot result in exposure of the FL that would lead to the increased attachment of TBEV to target cells.

In conclusion, chFVN145 demonstrated clear therapeutic efficacy, which was TBEV dose-dependent. ChFVN145 protected mice infected with TBEV-Eu, TBEV-Sib, and TBEV-FE strains. Importantly, administration of chFVN145 did not enhance TBEV infection in the mouse model. The neutralizing epitope was localized in domain III of glycoprotein E; sites responsible for binding with chFVN145 are distant from the FL, and their interaction with chFVN145 would not result in rearrangement of glycoprotein E and exposing the FL, which would lead to enhanced TBEV infection. The obtained results indicate that chFVN145 would be of value in designing potential anti-TBE preparations; however, further *in vivo* efficacy studies using multiple administrations of the antibody are required.
